# Infecções de Dispositivos Cardíacos Eletrônicos Implantáveis – Uma Realidade Crescente e Preocupante

**DOI:** 10.36660/abc.20210151

**Published:** 2021-06-08

**Authors:** Eduardo Arrais Rocha, João Lins de Araújo, Ricardo Pereira Silva

**Affiliations:** 1 Faculdade de Medicina Universidade Federal do Ceará FortalezaCE Brasil Faculdade de Medicina da Universidade Federal do Ceará , Fortaleza , CE - Brasil; 2 Centro de Arritmia do Ceará FortalezaCE Brasil Centro de Arritmia do Ceará , Fortaleza , CE - Brasil; 3 Universidade Federal do Ceará Hospital Universitário Walter Cantídio FortalezaCE Brasil Universidade Federal do Ceará - Hospital Universitário Walter Cantídio , Fortaleza , CE - Brasil

**Keywords:** Endocardite, Sepses, Marca-passos, Desfibriladores Cardíacos, Ecotransesofágico, Tomografia por Emissão de Pósitrons

As taxas de infecção de dispositivos cardíacos eletrônicos implantáveis (DCEI) têm crescido, determinando a necessidade de um amplo debate sobre o assunto. Diversas razões podem justificar o ocorrido, como: maior número de dispositivos implantados nos últimos anos, maior envelhecimento da população, novas técnicas e equipamentos, com procedimentos mais complexos e prolongados. ^1^

Maciel e Silva ^2^ abordam esse tópico de maneira clara, objetiva, trazendo uma importante contribuição da literatura nacional, em expressiva casuística, confirmando esses achados preocupantes, assim como discutindo suas repercussões, considerando as elevadas taxas de morbimortalidade e os altos custos envolvidos, principalmente nos quadros de endocardites e sepses.

O trabalho apresenta dados novos em relação à evolução dos pacientes com cardiopatia chagásica e DCEI, mostrando não haver diferenças em relação às variáveis clínicas, laboratoriais ou prognósticas, quando esses apresentam infecções nos dispositivos. ^2^

O estudo, por ser retrospectivo, apresenta algumas limitações inerentes, tanto as citadas no texto quanto o fato de terem sido incluídos pacientes de diferentes épocas, com terapêuticas distintas, envolvendo até as mais modernas técnicas de extração de eletrodos. Tal fato mereceria uma comparação das taxas de eventos em cada ano para avaliação do impacto dos novos conhecimentos adquiridos e das novas técnicas utilizadas no tratamento desta grave complicação.

O uso de novas técnicas diagnósticas, como os exames de imagem (tomografia de emissão de pósitrons – PET-CT, tomografia computadorizada cardíaca e cintilografia miocárdica com leucócitos marcados) para auxílio do diagnóstico de infecção dos eletrodos e visualização de suas complicações, como embolias inesperadas ou infecções metastáticas, têm crescido bastante na literatura, o que não pôde ser expressado no presente trabalho. ^3 , 4^

A ecocardiografia intracardíaca tem se mostrado útil em alguns cenários, possibilitando a biópsia da massa, podendo auxiliar o diagnóstico diferencial entre trombo e vegetação. ^1^ Entretanto, o ecocardiograma transesofágico mantém-se como principal exame de imagem no auxílio diagnóstico e de conduta, devendo ser repetido após uma semana, quando inicialmente negativo.

Considerando o cenário de aumento de procedimentos e complicações, diversos aspectos devem ser rigorosamente analisados e seguidos, como: a necessidade de técnicas cirúrgicas apuradas, com ampliações da loja dos DCEI, principalmente nas trocas; a utilização de implantes submusculares, evitando ou minimizando as extrusões dos geradores; o uso de rigorosa técnica de assepsia e hemostasia; o uso de técnicas e fios de suturas adequados e a realização de procedimentos em ambiente cirúrgico, com perfeitas condições assépticas, muitas vezes não disponíveis nas usuais salas de hemodinâmica, onde ocorre a grande parte dos implantes. A antibioticoprofilaxia com dose única no início da cirurgia segue como medida efetiva nas diretrizes. ^1 , 5 , 6^

Situações muito comuns como implantes em pacientes crônicos, com cateteres de diálise, com cateteres centrais, marca-passos provisórios, principalmente com tempo prolongado e implantados às vezes em situações de urgência, pacientes com tempo hospitalar prolongado, em unidades de terapia intensiva, com demora para o implante, às vezes por questões relacionadas à autorização e liberação da prótese, precisam urgentemente ser rediscutidas e resolvidas pelas diversas entidades envolvidas.

A gravidade dos doentes que estão sendo submetidos aos implantes também precisa ser repensada, principalmente em procedimentos eletivos e de prevenção primária. Pela própria gravidade da doença, muitos não terão tempo suficiente para benefício de um implante de DCEI preventivo, como no caso dos cardiodesfibriladores implantáveis.

A necessidade de equipes multidisciplinares para tratamento desta grave patologia (“ *endocarditis team* ”) é de extrema importância, com envolvimento do especialista em estimulação cardíaca, infectologista, microbiologista, radiologista, intensivista, internista, considerando que a implementação de um diagnóstico etiológico preciso e terapêutica apropriada é fundamental. ^7^

A identificação microbiológica do germe muitas vezes necessita de técnica de semeio mais prolongada, para germes atípicos e de crescimento lento, com maior número de amostras (>3 amostras) e repetição das coletas com intervalos maiores, permitindo a instituição de uma terapêutica antibiótica direcionada para os patógenos identificados. O tempo inadequado de terapia antibiótica e principalmente a não retirada completa do sistema, tem levado à maior taxa de recorrências e de morbimortalidade. ^1 , 7 - 9^

O time de especialistas permitirá uma discussão conjunta dos profissionais e da família, visando uma rápida decisão sobre a retirada do sistema, com planejamento sequencial sobre o novo implante, nunca devendo, entretanto, ser considerada a possibilidade de não o fazer, como em situações muito específicas. Talvez de todos os aspectos mencionados, o avanço das técnicas de extração e a experiência das equipes sejam os mais importantes a serem considerados dentro da realidade nacional. As sociedades nacionais precisam se mobilizar nesse sentido. Após a decisão de novo implante, deve-se considerar, quando disponível, o uso dos dispositivos subcutâneos como os desfibriladores e os marca-passos sem eletrodos, que têm demonstrado menores taxas de infecções, principalmente de endocardite e sepse. ^1 , 9 - 11^

As diversas diretrizes e estudos vigentes já publicados ^1 , 5 , 6 , 8 , 11 - 13^ deveriam servir para uniformizar e organizar as condutas, o que levaria a uma menor taxa de complicações e mortalidade. A SOBRAC – Sociedade Brasileira de Arritmias Cardíacas – está finalizando suas diretrizes em 2021, com amplo capítulo sobre o tópico, que ajudará bastante na resolução dessas questões, tendo também a Sociedade Latino-Americana de Ritmo Cardíaco (LAHRS) participado ativamente da recente diretriz. ^1^

Dentro desses aspectos citados, Maciel e Silva permitiram à comunidade científica uma ampla discussão sobre o assunto, com a riqueza de dados do trabalho, e gerou a necessidade de padronizações das sociedades locais e nacional, visando à monitorização e redução das taxas de infecção e suas graves consequências associadas.
