# Supplementary material for: Infecções de Dispositivos Cardíacos Eletrônicos Implantáveis – Uma Realidade Crescente e Preocupante
Source: Arq Bras Cardiol. 2021 Jun 8;116(6):1089–90. [Article in Portuguese] doi: 10.36660/abc.20210151 (PMC8288539; doi:10.36660/abc.20210151)
Supplement: Translation

# Translation: Infections of Cardiac Implantable Electronic Devices – A Growing, Worrying Reality

**Authors:** Eduardo Arrais Rocha, João Lins de Araújo, Ricardo Pereira Silva

**Affiliations:** 1 Brazil Faculdade de Medicina da Universidade Federal do Ceará, Fortaleza, CE - Brazil; 2 Brazil Centro de Arritmia do Ceará, Fortaleza, CE - Brazil; 3 Brazil Universidade Federal do Ceará - Hospital Universitário Walter Cantídio, Fortaleza, CE - Brazil

**Keywords:** Endocarditis, Sepsis, Pacemakers, Cardiac Defibrillators, Ecotransesophageal, Positron Emission Tomography

Infection rates of Cardiac Implantable Electronic Devices (CIEDs) have been increasing, determining the need for a wide debate on the subject. Several reasons can justify the issue, such as: a greater number of devices implanted over the latest years, overaging of the population, new techniques and equipment, with more complex and prolonged procedures.^1^

Maciel and Silva^2^ address this topic in a clear, objective manner, bringing an important contribution from the national literature, in a significant case selection, confirming those worrying findings and discussing their impacts, considering the high rates of morbidity and mortality and high costs involved, particularly in the cases of endocarditis and sepsis.

The work presents new data regarding the evolution of patients with Chagas’ heart disease and CIED, showing that there are no differences in relation to clinical, laboratory or prognostic variables, when their devices are shown to have infections.^2^

As a retrospective study, it has some inherent limitations, well referred to in its content, such as the inclusion of patients from different periods, under varied therapeutic approaches, including even the most cutting-edge electrode extraction techniques. Such fact should deserve an yearly comparison of the event rates in order to assess the impact of the latest knowledge acquired and the new techniques used in the treatment of such a severe complication.

The use of new diagnostic techniques, such as imaging exams (Positron Emission Tomography with Computed Tomography - PET/CT, Cardiac Computed Tomography and Myocardial Scintigraphy with marked leukocytes) to aid in the diagnosis of infection of the electrodes and visualization of their complications, such as unexpected embolisms or metastatic infections, has grown considerably in the literature, which could not be expressed in the present study.^3 , 4^

Intracardiac echocardiography has been shown to be useful in some scenarios, enabling mass biopsy, which may assist in the differential diagnosis between thrombus and vegetation.^1^ However, transesophageal echocardiography remains the main imaging test for diagnostic and conduct assistance, and should be repeated after one week, when initially negative.

Considering the scenario of increased procedures and complications, several aspects must be thoroughly analyzed and followed, such as: the need for refined surgical techniques, with expanded DCEI store availability, chiefly for exchanges; the use of submuscular implants, avoiding or minimizing extrusions of generators; the use of rigorous aseptic and hemostatic techniques; the use of appropriate suture stitching techniques and the performance of procedures in a surgical environment, with perfect aseptic conditions, often unavailable in the usual hemodynamic rooms, where most of the implants occur. Single-dose antibiotic prophylaxis at the beginning of surgery remains an effective measure in the guidelines.^1 , 5 , 6^

Very common situations such as implants in chronic patients, with dialysis catheters, central catheters, temporary pacemakers, particularly those the time of which was prolonged and sometimes implanted in urgent situations, patients with prolonged hospital stay, in intensive care units, facing delay for implantation, sometimes due to issues related to the authorization and release of the prosthesis, they urgently need to be discussed and resolved by the various entities involved.

The severity of patients who are undergoing implants also needs to be rethought, particularly in elective and primary prevention procedures, since due to the severity of the disease, many will not have enough time to benefit from a preventive CIED implant, as in the case of implantable cardiodefibrillators.

The need for multidisciplinary teams to treat this severe pathology (“endocarditis team”) is extremely important, with the involvement of a specialist in cardiac stimulation, infectious disease, microbiologist, radiologist, intensivist, internist, considering that the implementation of an accurate etiological diagnosis and appropriate therapeutic approach is paramount.^7^

The microbiological identification of the germ often requires a longer sowing technique, for atypical and slow-growing germs, with a greater number of samples (> 3 samples) and repetition of the collections with greater intervals, thus allowing an antibiotic therapy directed to the pathogens identified. The inadequate duration of antibiotic therapy and especially the failure to completely remove the system has led to a higher rate of recurrences and morbidity and mortality.^1 , 7 - 9^

The team of specialists will allow a joint discussion of the professionals and the family, aiming at a quick decision on the removal of the system, with sequential planning on the new implant, however, the possibility of not doing so should ever be considered, as in very specific situations. Perhaps, out of all the aspects mentioned, the advancement of extraction techniques and the experience of the teams are the most important elements to be considered within the national reality. National societies need to mobilize in this regard. After the decision for a new implant, the use of subcutaneous devices, such as defibrillators and pacemakers without electrodes, should be considered when available, which have shown lower rates of infections, mainly of endocarditis and sepsis.^1 , 9 - 11^

The various guidelines and current studies already published^1 , 5 , 6 , 8 , 11 - 13^ should serve to standardize and organize the conduct, which would lead to a lower rate of complications and mortality. SOBRAC - Brazilian Society of Cardiac Arrhythmias is finalizing its guidelines in 2021, with a broad chapter on the topic, which will help a lot in the resolution of these issues, and the Latin American Society of Cardiac Rhythm (LAHRS) has also actively participated in the recent guideline.^1^

Within these aspects mentioned, Maciel and Silva allowed the scientific community to have a wide discussion on the subject, with the wealth of data on the work and generated the need for standardization of local and national society, aiming at monitoring and reducing infection rates and their serious associated consequences.
